# Expression of *Drosophila melanogaster* V-ATPases in Olfactory Sensillum Support Cells

**DOI:** 10.3390/insects15121016

**Published:** 2024-12-22

**Authors:** Kalpana Jain, Sinisa Prelic, Bill S. Hansson, Dieter Wicher

**Affiliations:** Department Evolutionary Neuroethology, Max Planck Institute for Chemical Ecology, 07745 Jena, Germany; kjain@ice.mpg.de (K.J.); sprelic@ice.mpg.de (S.P.); hansson@ice.mpg.de (B.S.H.)

**Keywords:** proton transmembrane transport, olfaction, antenna, OSN, support cell, sensillum lymph, H^+^ homeostasis

## Abstract

V-ATPases are ubiquitous and evolutionarily conserved rotatory proton pumps, which are crucial for maintaining various biological functions. Previous investigations have shown that a V-ATPase is present in the support cells of moth trichoid sensilla and influences their olfactory sensory neuron performance. Generally, V-ATPases are thought to regulate the pH value within the sensillum lymph, and aid K^+^ homeostasis within the sensillum. This, in turn, could influence various mechanisms involved within the support cells, like maintaining the receptor membrane potential (receptor current), nutrient and ion transport, odorant solubility, and various signaling mechanisms. In this study, we identify V-ATPase expression and localization in the *Drosophila melanogaster* antenna using bioinformatics and immunohistochemistry. Elucidating an olfactory V-ATPase function will improve our current understanding of how support cells contribute to *Drosophila*’s sense of smell.

## Simple Summary

Many cellular processes in eukaryotic cells are regulated by vacuolar (H^+^)-ATPases (V-ATPases), energy-driven proton pumps that primarily function to acidify intracellular organelles across all eukaryotes. These enzymes are densely packed on both the plasma membrane and the endomembrane in specific cell types of insects and vertebrates. Significant prior studies have demonstrated the presence of V-ATPases in insect olfactory sensilla, highlighting their role in olfaction. In our research, we used bioinformatics and immunohistochemistry to investigate the expression and localization of V-ATPase in the *Drosophila melanogaster* antenna. Our findings show that genes encoding V-ATPase are highly expressed in the *Drosophila* antenna, as demonstrated by bulk and single-cell antennal transcriptome analyses. The results from immunohistochemistry further confirm that V-ATPase is restricted to non-neuronal support cells within the antenna. We therefore propose that V-ATPase activity in support cells plays a specific and important role in odor processing.

## 1. Introduction

Homeostasis, or the maintenance of stable internal conditions independent of changing external factors, is a fundamental requirement for living organisms [1,2]. Insects, for example, need to regulate their sensory sensitivity, respiration, body temperature, and movement to thrive in a constantly changing environment [3]. An important organelle that contributes to homeostasis at the cellular level is the mitochondrion, which generates adenosine triphosphate (ATP) through oxidative phosphorylation to provide energy for various cellular processes [4]. A molecule essential for cellular homeostasis is the H^+^ vacuolar-type ATPase (V-ATPase), an evolutionarily conserved protein that functions as a primary proton pump derived from a common ancestral enzyme [5,6,7,8]. V-ATPases create an electrochemical proton gradient across cell membranes, acidifying intracellular vesicles and organelles [9,10,11,12,13]. They also balance the cellular pH and contribute to the transport of ions and nutrients across membranes, thereby regulating protein sorting and degradation [14,15,16,17,18,19,20]. Such proton pumps are often found in the apical regions of eukaryotic cells [5,21] and are involved in various signal transduction pathways such as mTOR, Notch, and Wnt [22]. For instance, V-ATPases play roles in synaptic vesicle acidification, vital for neurotransmitter uptake and release in neurons [23].

Earlier researchers identified a so called “K^+^ pump” in various insect epithelial tissues, which is now known as V-ATPase [24,25,26,27,28,29]. V-ATPases are expressed in insect organs like the salivary glands of the *Calliphora* blowfly, Malpighian tubules of the blood-sucking bug *Rhodnius*, the midgut of moths, and various insect sensory organs [30]. V-ATPases also occur in endomembrane and apical plasma membranes of insect epithelia [30]. For example, V-ATPase monoclonal antibodies have been previously employed to detect V-ATPase protein in an extremely folded apical plasma membrane region of support cells of moth sensilla [31,32]. Earlier, it was shown that these electrogenic potassium pumps (H^+^ V-ATPase) were responsible for driving receptor currents upon ligand–receptor interaction and act as an energy source for transepithelial voltage maintenance [33]. These pumps create a proton gradient across the membrane by pumping H^+^ ions into cells. Another protein, the K^+^/H^+^ antiporter, uses this H^+^ gradient to transport K^+^ into the cell [28,30,34,35]. The K^+^ transport mechanism has been observed in many insect tissues including the Malpighian tubules of the yellow fever mosquito *Aedes aegypti* and in the support cells of mechanosensory sensilla of the cockroach *Periplaneta americana* [36,37]. Several genes coding for V-ATPase subunits have since been identified in the *Drosophila* genome with high expression levels in the Malpighian tubules, rectum, antennal palps, and uterus [34,38,39]. The functional disruption of V-ATPase-mediated acidification has been shown to cause aberrant signaling during *D. melanogaster* and *Caenorhabditis elegans* embryogenesis [38,40].

The present study seeks to determine whether a V-ATPase might play a role in *Drosophila* olfaction. *D. melanogaster* possesses two olfactory appendages on their heads: the antennae and maxillary palps [41,42,43]. Of these, the third antennal segment is most prominently accountable for olfaction, and is covered with chemosensory hair-like structures termed sensilla [41,43,44]. These sensilla house the olfactory sensory neurons (OSNs) responsible for transducing odor information. Crucially, OSNs are enveloped by three different types of supporting cells: the thecogen cell (first layer), trichogen cell (second layer), and tormogen cell (outermost layer) triad [44,45]. OSN dendrites are combinatorially equipped with families of olfactory receptors such as odorant receptors (ORs) and ionotropic receptors (IRs) [46,47,48,49]. These proteins are composed of a complex of an odor-binding protein granting odor specificity, and a highly conserved co-receptor protein(s) according correct regulation, trafficking, and receptor functioning, thereby forming ligand-gated cation channels that transduce odors into neuronal signals [49,50,51,52,53,54,55,56]. Crucially, all OSN dendrites are surrounded by a K^+^ rich sensillum lymph [33]. A previous study has indicated an active role of support cells in *D. melanogaster* odor processing [45]; specifically, thecogen cells appear to take up K^+^ released by OSNs during odor transduction [45]. As V-ATPases and K^+^/H^+^ antiporters are likely to form a K^+^ uptake system together, we investigated whether V-ATPases might occur in the olfactory cells in the *D. melanogaster* antenna. Supporting this idea, a previous study documented abundant V-ATPase expression in the tormogen cells of the trichoid sensilla of male moths [31,57]. This study aims to update our understanding of antennal V-ATPases using molecular methodologies to elucidate where V-ATPase is expressed in the antenna of the *D. melanogaster* model organism using bioinformatics and immunohistochemistry.

## 2. Materials and Methods

### 2.1. Bioinformatic Analysis

Thirty-six genes encoding all *Drosophila* V-ATPase subunits (FlyBase gene group FBgg0000111) were shortlisted for gene expression comparison, with other members annotated with the Gene Ontology term “proton transmembrane transport” (GO:1902600) as a functionally related control set. Eight antennal reference genes (*Orco*, *Ir8a*, *Ir25a*, *elav*, *repo*, *nompA*, *Su(H)*, *sv*) were selected based on involvement as common antennal cell type markers (as three coreceptors demarcating various OSN classes, and as neuronal, glial, thecogen- and tormogen-specific, and support-cell-demarcating markers, respectively). Ubiquitous, pancellular housekeeping genes were further selected as transcriptional references for involvement in common cytoskeletal, metabolic, physiological, and enzymatic functions (*Act5C*, *Gapdh1*, *Cam*, and *eEF1β*, respectively). For bioinformatic analysis, we consulted two separate bulk antennal tissue RNA-seq transcriptomic datasets. One set compares adult male and female *D. melanogaster*, sampling 1200 antennae pairs for each sex in Canton-S flies aged >1 day old post-eclosion [58]. The second RNA-seq dataset pools 300 mixed sex *D. melanogaster* of 5–12 day old age, for wildtype Canton-S flies and homozygous *atonal* (*ato*) mutants [59].

Fragments/reads per kilobase per million mapped fragments/reads (FPKM or RPKM) values for all genes detectable in the antenna of each fly cohort were ordered, plotted, labeled, and color-coded using R (version 4.2.0). For all studies, all (outdated, synonymous, or alternative) gene names/symbols were converted to the FlyBase standard gene symbol for parity, and checked manually and automatically with a custom script. All gene nomenclature follows FlyBase gene symbol naming conventions. Gene transcripts are italicized and references to protein counterparts are unitalicized in line with FlyBase nomenclature guidelines.

For single-cell data, we consulted the antennal “10x stringent” dataset of the *Fly Cell Atlas* derived using a microfluidic droplet-based cell capture methodology [60]. Differential expression analysis was performed comparing cell group classifications on specific gene expression using non-parametric Wilcoxon rank-sum statistical testing (via Seurat) using the Automated Single-cell Analysis Pipeline (ASAP) portal asap.epfl.ch [61], with the following parametrization: *minimum % of cells with gene > 0 = 0.1* (10%); *min%diff = NULL*; *max cells per group = NULL*; *foldchange cutoff* was not used to prevent any data subsetting. Explicit parametrization settings and the data source list have been made available in the data repository associated with this study. A false detection rate (FDR)-adjusted *p*-value of <0.05 was used to differentiate significantly up- or downregulated genes for each cell type class, defined based on the grouping “*annotation_broad*”.

### 2.2. Fly Genetics, Immunohistochemistry, and Microscopy

In this stage, 6–8-day-old transgenic *D. melanogaster* flies were used for all immunohistochemistry and imaging. The GAL4-UAS system was used to label thecogen and tormogen support cells using nompA-GAL4 and ASE5-GAL4 lines, respectively (kindly provided by Craig Montell (University of California, Santa Barbara, CA, USA)). nompA-GAL4 and ASE5-GAL4 fly lines were crossed with UAS-mCD8::GFP [62] to visualize support cells via a fluorescent reporter. N-GFP-Orco [63] and UAS-DenMark fly lines were used to label OSNs [64,65]. Here, the GAL4-UAS system was used to generate transgenic fly lines expressing the fluorescent dendritic marker reporter DenMark or chimeric N-GFP-Orco in OSNs in an Orco-null genetic background (Orco-GAL4, orco^1^). These fly lines were used for subsequent cryosectioning, immunostaining of antennae, and confocal fluorescence imaging. First, 60 antennae from 30 flies were dissected and mounted in an optimal cutting temperature (OCT) mounting media (Lot: 03816567; VWR Chemicals, Leuven, Belgium). Microm HM 560 was used to cut thin antennal sections on FisherSuperFrost Plus slides. The sections were then immediately fixed using 4% paraformaldehyde for 10 min in a humidified chamber. Afterwards, the slides were washed twice using phosphate-buffered saline (PBS) for 10 min on a shaker at room temperature. The slides were then kept in blocking solution (5% normal goat serum in PBS) for 30 min and covered with a coverslip (#1). The blocking solution was then removed and primary monoclonal antibody was added prior to covering slides with a coverslip (#1), and left for overnight incubation at 4 °C. The next day, the slides were washed four times with PBS for 10 min each, and then blocked with blocking solution for 30 min. Following this, secondary fluorescent antibodies were added to each slide and incubated for two hours at room temperature in the dark. Later, the slides were washed four times, for five mins per wash, in PBS on a shaker in the dark. Finally, Vectashield (Vector Laboratories, Newark, CA, USA) was added onto the slides, which were then covered with a coverslip (#0) for storage at 4 °C. Primary antibodies used: ‘47-5’ mouse monoclonal antibody raised against the G (16 kDa) and E (28 kDa) non-catalytic subunits of the V1 complex of purified larval midgut V-ATPase of *Manduca sexta* [31,32] (3:25 dilution) (kindly gifted by Hans Merzendorfer and Helmut Wieczorek); chicken anti-GFP (1:500 dilution) (Invitrogen, Carlsbad, CA, USA); and anti-Orco (1:1000 dilution) (kindly provided by Leslie Vosshall (Rockefeller University, New York, NY, USA)). Secondary fluorescent antibodies used: goat anti-mouse Alexa 546 (1:250 dilution); goat anti-chicken Alexa 488 (1:250 dilution); goat anti-rabbit Alexa 488 (1:250 dilution); goat anti-mouse Alexa 633 (1:250 dilution). All secondary antibodies were purchased from Invitrogen. Imaging of the antennal sections was performed using a Zeiss cLSM880 confocal microscope (Carl Zeiss, Oberkochen, Germany) with a 10× and 40×/1.20 water immersion objective (C-Apochromat, Carl Zeiss). The samples were excited with 488 nm, 543 nm, and 633 nm wavelengths for different experiments. The laser and the detector gain for two channels were optimized to avoid saturation and all images were acquired with the same confocal settings for contrast and brightness. Z-stack images were captured at 2048 × 2048-pixel resolution at an 8-bit color depth and presented as maximum intensity projections.

## 3. Results and Discussion

**Figure 1:**
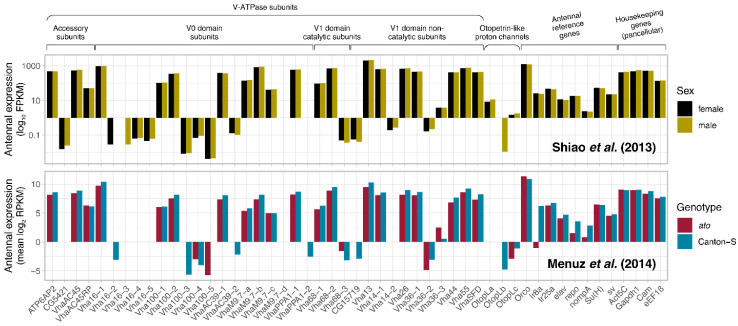
Bulk antennal tissue expression of *Drosophila* V-ATPase subunit and other genes with known involvement in proton transmembrane transport, alongside antennal and housekeeping reference genes. Myriad V-ATPase genes are highly abundant in expression in various antennal tissues, including all functional subunits. Two antennal RNA-seq datasets show contrasting expression across fly sex [58] and wildtype Canton-S vs. *ato* mutant flies lacking coeloconic sensilla of the IR olfactory subsystem [59].

To first gauge whether V-ATPase is expressed in the *Drosophila* antenna, we consulted two independent antennal transcriptomes of *D. melanogaster*, comparing the expression of all known *Drosophila* V-ATPase subunit transcripts to a reference panel of antennal and pancellular housekeeping genes across two separate studies comparing female and male antennae [58], and the antennae of the wildtype Canton-S strain and *atonal* (*ato*) mutants lacking coeloconic sensilla, which constitute the IR olfactory subsystem [59]. Here, we considered all 36 known genes involved in the V0 and V1 (both catalytic and non-catalytic) domains of V-ATPase and its accessory subunits [38,66,67]. We found that a large majority of these subunit transcripts are highly expressed in both datasets, with 20 of 36 genes at degrees of expression comparable to reference gene levels, in both studies, including all structural subunit types (Figure 1).

**Figure 2:**
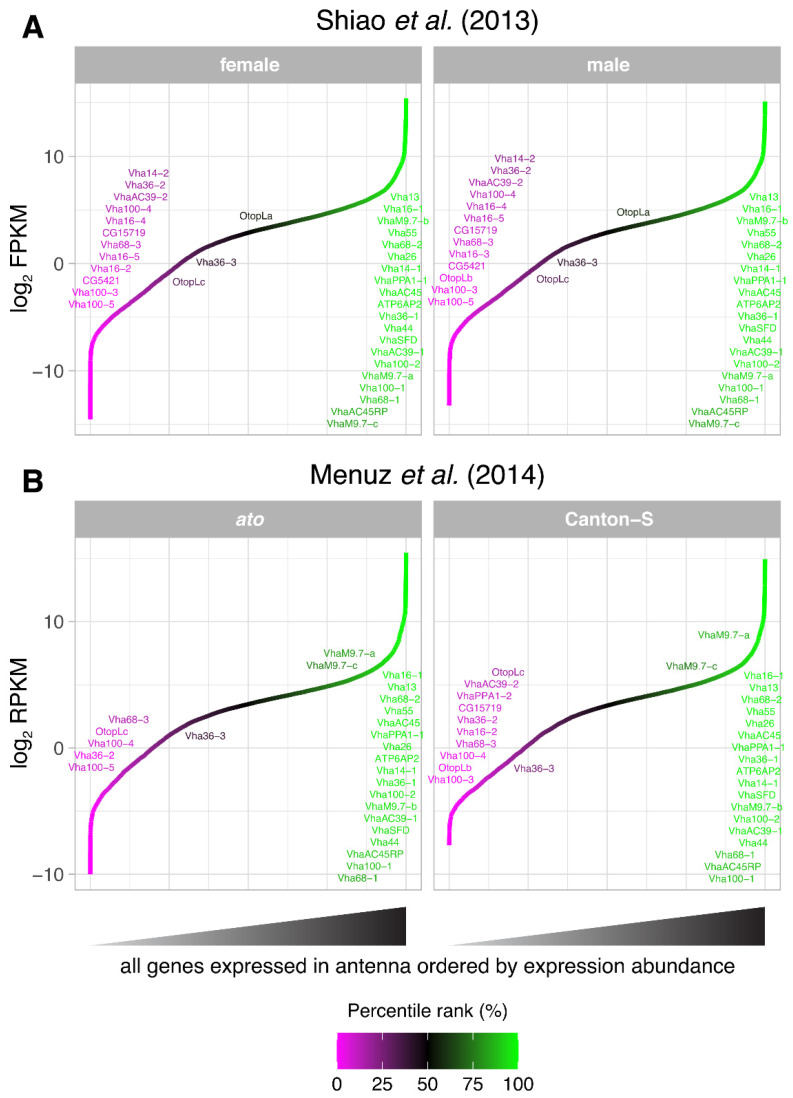
V-ATPase subunit expression abundance in various antennal transcriptomes. Expression of all genes detected in the *Drosophila* antennal datasets [58,59] is plotted, colored, and ordered low-to-high by its expression abundance. All known genes involved in proton transmembrane transport (including all V-ATPase subunits) are labeled, whereby labels are vertically displaced from where the gene appears. V-ATPase subunit expression is compared across fly sex (**A**) and between fly cohorts with intact or absent coeloconic sensilla constituent of the IR olfactory subsystem (**B**).

Next, to understand how abundant V-ATPase subunits are among antennal genes, we plotted the expression of all antennal genes detected within each antennal transcriptome, ordered by expression level abundance. In transcriptomes from both studies, we found that 20 common V-ATPase subunits rank among the most abundant antennal genes by expression (83rd–100th or 76th–100th percentile range in each respective study) (Figure 2). We found expression levels and percentile rankings of these abundant V-ATPase subunits to corroborate well between both studies. Depending on the experimental condition, a further 13 V-ATPase subunits were detectably expressed in one dataset (within the 0th–40th percentile ranges) (Figure 2A), and 5 and 9 subunits in the other (0th–38th or 0th–25th percentile range for *ato* or Canton-S fly cohorts, respectively) (Figure 2B). Furthermore, we found no sexually dimorphic (sex-biased) expression for any gene involved in proton transmembrane transport (FDR < 0.05) [58]. Only minor differences were found between *ato* mutant vs. wildtype flies regarding V-ATPase subunit expression: a 1.44- to 1.71-fold depletion exists for *VhaSFD*, *VhaM9.7-b*, *Vha26*, *Vha44*, *Vha13*, *Vha16-1*, and *Vha100-2* above the detection threshold (FDR < 0.05) [59]. Though this initially might suggest the enrichment of V-ATPase subunits within a particular (coeloconic) sensillum type, data from a similar study comparing antennal expression between wildtype flies and *absent MD neurons and olfactory sensilla* (*amos*) mutants that lack basiconic and trichoid sensilla (constituent of the OR olfactory subsystem) reveal that 15 subunits (including all 7 aforementioned subunits) also appear o be depleted in the *amos* mutant antennal transcriptome at the same FDR threshold (see data repository) [68]. This likely suggests that the loss of any sensillum type or olfactory subsystem contributes to V-ATPase subunit transcript depletion, and, thus, that V-ATPase subunits are generic components of all antennal sensilla rather than residing elsewhere in the antennal organ.

Taken together, the bulk transcriptomic analyses suggest that most *Drosophila* V-ATPase subunits are present in the antenna; that a majority subset of these subunits is highly abundant in this tissue, likely constituent within sensilla; and that their expression is neither sex- nor olfactory-subsystem-biased.

**Figure 3:**
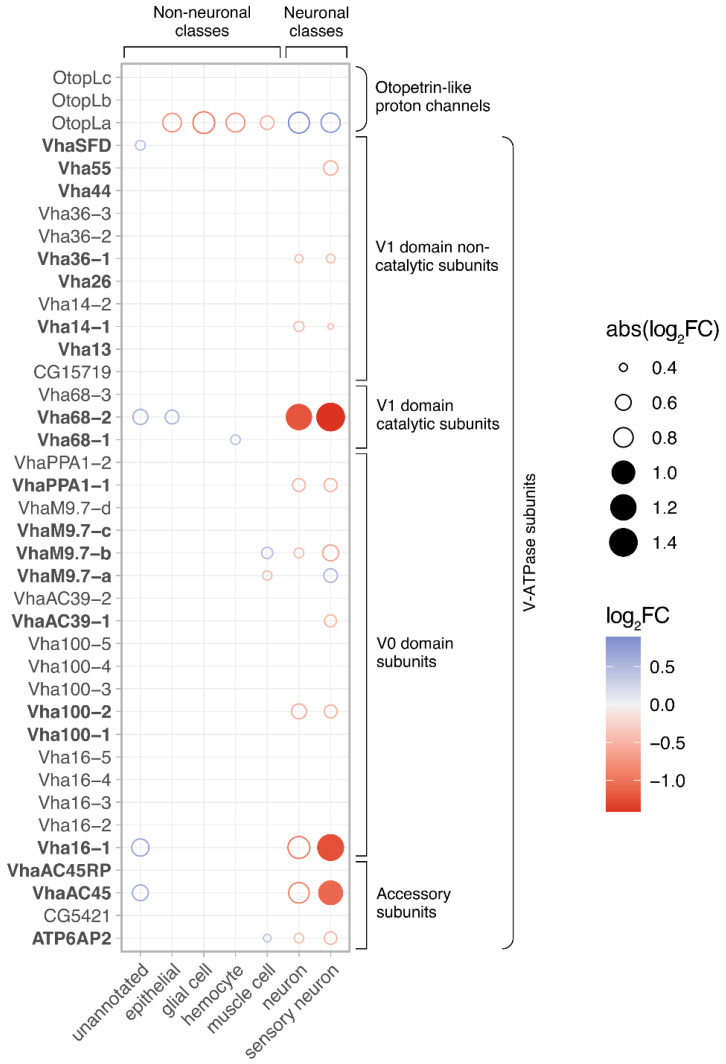
Differential expression of all *Drosophila* genes with known involvement in proton transmembrane transport (including all V-ATPase subunits) in the *Fly Cell Atlas* single-cell antennal transcriptome. Gene expression for each annotated cell type class is compared with all other antennal cells using a Wilcoxon test. Circles are plotted only where significant differential gene expression is detected for a cell type class (FDR < 0.05), colored by relative up- (blue) or downregulation (red) and sized based on absolute expression difference magnitude. Circles are filled only when gene expression difference is larger than a two-fold difference in expression (|log2FC| > 1). Genes in bold have been previously identified as highly abundant in antennal tissue. FC: fold change.

We next wanted to infer the localization of V-ATPases bioinformatically, by assessing whether any V-ATPase subunit transcripts segregate differentially among antennal cell types. Here, we consulted the antennal single-cell transcriptomes of the *Fly Cell Atlas* [60] and performed a differential expression analysis checking whether any V-ATPase subunits appear preferentially enriched or depleted in certain annotated classes (i.e., antennal cell type groupings). We recorded significant detections (FDR < 0.05 threshold) using a bubble plot showing the magnitude of depletion/enrichment for all V-ATPase subunit gene transcripts across all cell type classes. Here, we found a general pattern wherein many antennal V-ATPase subunits were depleted in neuronal cell types, but not in non-neuronal and unannotated classes (Figure 3), with the latter largely consisting of non-neuronal cells. On the other hand, though fewer, we found several cases of ATPase subunit enrichment in non-neuronal cell classes (Figure 3), indicating that V-ATPases are likelier found within non-neuronal cell populations of the antenna. For comparative purposes, inverse to this pattern, a single proton transmembrane transporter *Otopetrin-like c* (*OtopLc*), not a constituent of the V-ATPase complex [69], was found specifically enriched in neuronal classes and depleted in non-neuronal cell classes (Figure 3). We therefore conclude V-ATPase subunits, specifically among proton transmembrane transporters, are likely to be localized to non-neuronal cells of the *Drosophila* antenna.

**Figure 4:**
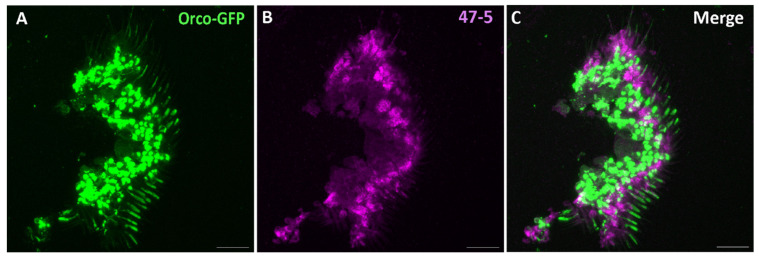
Expression pattern of V-ATPase in the antennal cross sections of N-GFP-Orco *D. melanogaster* fly line. (**A**) Native GFP signal from the N-GFP-Orco fly. (**B**) Immunofluorescence micrographs of V-ATPase (47-5, magenta) staining of the same antennal cross sections shown in (**A**). (**C**) No colocalization of GFP and V-ATPase observed in compartments of *Drosophila* OSNs. Scale bar: 17 µm.

**Figure 5:**
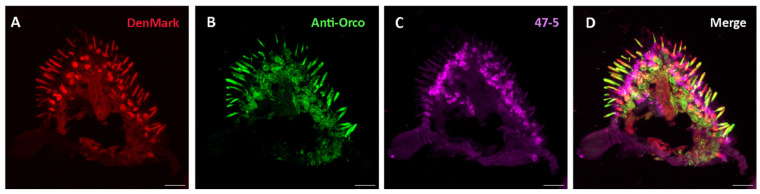
Expression pattern of V-ATPase in the antennal cross sections of DenMark-expressing *D. melanogaster* fly line. Native fluorescence of DenMark (red) (**A**), Orco-positive OSN immunofluorescence (green) (**B**), V-ATPase protein immunofluorescence (47-5, magenta) (**C**). (**D**) Absence of V-ATPase in olfactory sensory neurons and colocalization of DenMark and anti-Orco in these OSNs. Scale bar: 17 µm.

Subsequently, at the protein level, we wanted to check whether V-ATPase might be localized in *Drosophila* antennal OSNs in preparations of cryosectioned antennae. To this end, we used transgenic N-GFP-Orco flies expressing GFP conjugated to Orco chimerically, shown previously to label Orco-positive OSNs [63], along with the ‘47-5’ mouse monoclonal antibody raised against purified V-ATPase from the *Manduca* midgut [31,32]. Specifically, this antibody’s epitopes are the G (13 kDa) and E (26 kDa) subunits of the V1 complex of V-ATPase, which correspond to the products of the *Vha13* and *Vha26* transcripts [34,67,70], both previously found abundantly in *Drosophila* antennae (Figure 1 and Figure 2). In a previous immunocytochemical imaging study, this antibody was found to bind to distinct V-ATPase subunits in the sensilla of *Manduca sexta* and *Anteraea pernyi* moths [31]. Following immunohistochemical preparation, we observed some co-localization of native N-GFP-Orco and V-ATPase signal in some OSNs (Figure 4). However, we suspected this might be artifactual, since the vast majority of OSNs did not exhibit co-localization with V-ATPase. To clarify this, we further utilized DenMark, a hybrid protein composed of red fluorescent protein mCherry and mouse protein ICAM5/Telencephalin [64]. DenMark was chosen because it is highly expressed in the soma and dendrites of multi-dendritic neurons in *Drosophila* larvae [64] and has been used successfully as a dendritic marker for *Drosophila* OSNs [65]. Therefore, we used the Orco-GAL4, orco^1^ driver line in combination with UAS-DenMark and UAS-N-GFP-Orco reporter lines to label all Orco-positive OSNs. We then performed immunohistochemistry using antibodies against Orco and V-ATPase in the antennae of flies. We found OSNs clearly labeled with a native DenMark signal; i.e., red fluorescence was directly visualized without any secondary antibody staining for DenMark. Here, we observed no V-ATPase co-localization with Orco nor DenMark (Figure 5). This suggests most (Orco-positive) OSNs are devoid of V-ATPase. Notably, from this inspection, we cannot definitively rule out that V-ATPase may be closely associated with OSNs at the inner dendrite or ciliary constriction junction. Furthermore, this imaging of Orco-positive OSNs does not rule out the possibility that V-ATPase might be expressed in a minority of *Drosophila* OSNs found in different sensillum types containing IR-expressing OSNs, or exist at the axonal termini of OSNs in the antennal lobes (central nervous system) beyond the antennal appendage. Relatedly, a previous immunofluorescence study reported that V-ATPase is present in all sensillum types within the support cells of the moth *Antheraea pernyi* [31].

**Figure 6:**
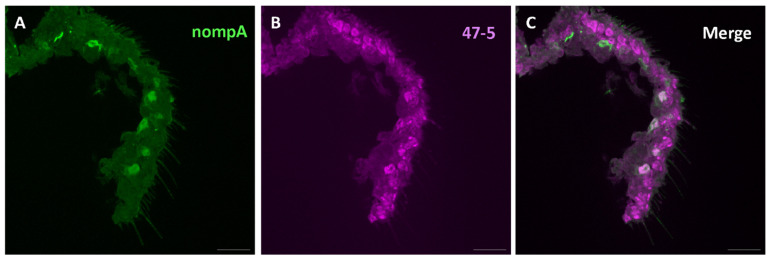
Expression pattern of V-ATPase in the thecogen cells of *D. melanogaster* antennal cross sections. (**A**) Immunofluorescence micrographs of mCD8::GFP (anti-GFP, green) and V-ATPase (47-5, magenta). (**B**) From the antennal cross sections of nompA-GAL4 fly line labeling thecogen cells. (**C**) Observation of partial colocalization of mCD8::GFP and V-ATPase in the thecogen cells. Scale bar: 17 µm.

**Figure 7:**
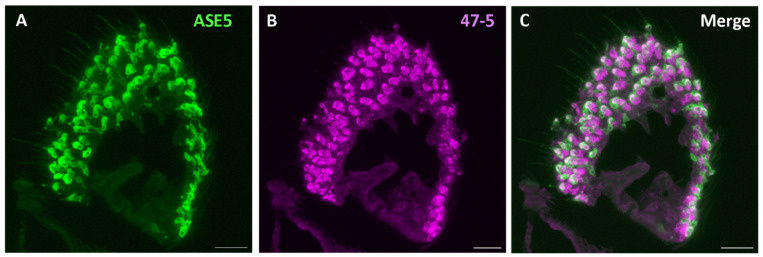
Expression pattern of V-ATPase in the tormogen cells of *D. melanogaster* antennal cross-sections. (**A**) Immunofluorescence image of mCD8::GFP (anti-GFP, green) and V-ATPase (47-5, magenta). (**B**) From the antennal cross sections of ASE5-GAL4 fly line labeling tormogen cells. (**C**) Observation of major colocalization of mCD8::GFP and V-ATPase in tormogen cells. Scale bar: 17 µm.

Given that V-ATPase is a ubiquitous proton pump essential for maintaining acidic environments within cellular compartments [30] and likely plays crucial roles in ion homeostasis at the level of the sensillum [26,32], we purport that V-ATPase activity within non-neuronal support cells specifically plays a crucial regulatory role in odor processing. We therefore checked whether V-ATPase is present in the support cells of *Drosophila* antennae. For this, we employed the available specific labeling of two of three support cell types of the sensillum: thecogen and tormogen cells. First, a nompA-GAL4 transgenic fly line was used, driving the expression of membrane-bound GFP specifically in thecogen cells [45,71]. Though few intact thecogen cells were present, we observed a typical co-localization with the V-ATPase antibody in these thecogen cells (Figure 6). Given the appearance of other cells labeled with the V-ATPase antibody, we next used the ASE5-GAL4 line to drive membrane-bound GFP expression in tormogen cells specifically [45,72]. Here, we found the more pronounced co-localization of V-ATPase protein in most observed tormogen cells (Figure 7). We also observed intermediate labeling, likely between thecogen and tormogen cells, which may suggest V-ATPase localization to trichogen cells, which are located between these two cell types. Because no specific method currently exists for labeling trichogen cells, we can only extrapolate that V-ATPase might be present in the trichogen cells of antennal sensilla (Figure 6 and Figure 7).

In conclusion, we show that a specific majority of transcripts encoding functional subunits of *Drosophila* V-ATPase are highly abundantly expressed in the *Drosophila* antenna in various antennal transcriptomes. Furthermore, both single-cell transcriptomic analyses and immunohistochemical co-labeling of V-ATPase and antennal cell types indicate that V-ATPase is largely restricted to the non-neuronal support cells of the antenna. Though V-ATPase appears to localize to thecogen and tormogen support cells, it remains unknown whether V-ATPase is explicitly expressed in trichogen, glial, or epithelial cells also found in the antennal appendage. Therefore, to understand the role of V-ATPase in *Drosophila* olfactory sensilla, further physiological investigations and expression studies are necessary to determine how V-ATPases contribute functionally to odor processing at the olfactory periphery of insects.

## Data Availability

All original data used in this study are archived in the Figshare online data repository at the following link: https://doi.org/10.6084/m9.figshare.27994004.

## References

[1] Cooper S.J. (2008). From Claude Bernard to Walter Cannon. Emergence of the concept of homeostasis. Appetite.

[2] Billman G.E. (2020). Homeostasis: The underappreciated and far too often ignored central organizing principle of physiology. Front. Physiol..

[3] Flavell S.W., Gogolla N., Lovett-Barron M., Zelikowsky M. (2022). The emergence and influence of internal states. Neuron.

[4] Brand M.D., Orr A.L., Perevoshchikova I.V., Quinlan C.L. (2013). The role of mitochondrial function and cellular bioenergetics in ageing and disease. Br. J. Dermatol..

[5] Eaton A.F., Merkulova M., Brown D. (2021). The H^+^-ATPase (V-ATPase): From proton pump to signaling complex in health and disease. Am. J. Physiol. Cell Physiol..

[6] Muench S.P., Trinick J., Harrison M.A. (2011). Structural divergence of the rotary ATPases. Q. Rev. Biophys..

[7] Nelson N. (1992). Evolution of organellar proton-ATPases. Biochim. Biophys. Acta Bioenerg..

[8] Forgac M. (1999). Structure and properties of the Vacuolar H^+^-ATPases. J. Biol. Chem..

[9] Kellokumpu S. (2019). Golgi pH, ion and redox homeostasis: How much do they really matter?. Front. Cell Dev. Biol..

[10] Colacurcio D.J., Nixon R.A. (2016). Disorders of lysosomal acidification—The emerging role of v-ATPase in aging and neurodegenerative disease. Ageing Res. Rev..

[11] Mindell J.A. (2012). Lysosomal Acidification Mechanisms. Annu. Rev. Physiol..

[12] Hnasko T.S., Edwards R.H. (2012). Neurotransmitter Corelease: Mechanism and Physiological Role. Annu. Rev. Physiol..

[13] Breton S., Brown D. (2013). Regulation of luminal acidification by the V-ATPase. Physiology.

[14] Casey J.R., Grinstein S., Orlowski J. (2010). Sensors and regulators of intracellular pH. Nat. Rev. Mol. Cell Biol..

[15] Paroutis P., Touret N., Grinstein S. (2004). The pH of the secretory pathway: Measurement, determinants, and regulation. Physiology.

[16] Maxfield F.R., McGraw T.E. (2004). Endocytic recycling. Nat. Rev. Mol. Cell Biol..

[17] Naslavsky N., Caplan S. (2018). The enigmatic endosome—Sorting the ins and outs of endocytic trafficking. J. Cell Sci..

[18] Forgac M. (2007). Vacuolar ATPases: Rotary proton pumps in physiology and pathophysiology. Nat. Rev. Mol. Cell Biol..

[19] Sun-Wada G.-H., Wada Y., Futai M. (2004). Diverse and essential roles of mammalian vacuolar-type proton pump ATPase: Toward the physiological understanding of inside acidic compartments. Biochim. Biophys. Acta Bioenerg..

[20] Hurtado-Lorenzo A., Skinner M., Annan J.E., Futai M., Sun-Wada G.-H., Bourgoin S., Casanova J., Wildeman A., Bechoua S., Ausiello D.A. (2006). V-ATPase interacts with ARNO and Arf6 in early endosomes and regulates the protein degradative pathway. Nat. Cell Biol..

[21] Nishi T., Forgac M. (2002). The vacuolar H^+^-ATPases—Nature’s most versatile proton pumps. Nat. Rev. Mol. Cell Biol..

[22] Sun-Wada G.-H., Wada Y. (2015). Role of vacuolar-type proton ATPase in signal transduction. Biochim. Biophys. Acta Bioenerg..

[23] Moriyama Y., Maeda M., Futai M. (1992). The role of V-ATPase in neuronal and endocrine systems. J. Exp. Biol..

[24] Ramsay J.A. (1953). Active transport of potassium by the malpighian tubules of insects. J. Exp. Biol..

[25] Wolfersberger M.G., Harvey W.R., Cioffi M., Kleinzeller A., Bronner F., Slayman C.L. (1982). Transepithelial Potassium Transport in Insect Midgut by an Electrogenic Alkali Metal Ion Pump. Current Topics in Membranes and Transport.

[26] Harvey W.R., Cioffi M., Wolfersberger M.G. (1983). Chemiosmotic potassium ion pump of insect epithelia. Am. J. Physiol..

[27] Harvey W.R., Maddrell S.H.P., Telfer W.H., Wieczorek H. (1998). H^+^ V-ATPases energize animal plasma membranes for secretion and absorption of ions and fluids. Integr. Comp. Biol..

[28] Wieczorek H., Putzenlechner M., Zeiske W., Klein U. (1991). A vacuolar-type proton pump energizes K^+^/H^+^ antiport in an animal plasma membrane. J. Biol. Chem..

[29] Wieczorek H., Grüber G., Harvey W.R., Huss M., Merzendorfer H., Zeiske W. (2000). Structure and regulation of insect plasma membrane H^+^ V-ATPase. J. Exp. Biol..

[30] Wieczorek H., Beyenbach K.W., Huss M., Vitavska O. (2009). Vacuolar-type proton pumps in insect epithelia. J. Exp. Biol..

[31] Klein U., Zimmermann B. (1991). The vacuolar-type ATPase from insect plasma membrane: Immunocytochemical localization in insect sensilla. Cell Tissue Res..

[32] Klein U., Löffelmann G., Wieczorek H. (1991). The midgut as a model system for insect K^+^-transporting epithelia: Immunocytochemical localization of a vacuolar-type H^+^ pump. J. Exp. Biol..

[33] Thurm U., Küppers J. (1980). Epithelial Physiology of Insect Sensilla. Insect Biology in the Future.

[34] Dow J., Davies S., Guo Y., Graham S., Finbow M., Kaiser K. (1997). Molecular genetic analysis of V-ATPase function in *Drosophila melanogaster*. J. Exp. Biol..

[35] Wieczorek H., Weerth S., Schindlbeck M., Klein U. (1989). A vacuolar-type proton pump in a vesicle fraction enriched with potassium transporting plasma membranes from tobacco hornworm midgut. J. Biol. Chem..

[36] Küppers J., Bunse I. (1996). A primary cation transport by a V-type ATPase of low specificity. J. Exp. Biol..

[37] Weng X.-H., Huss M., Wieczorek H., Beyenbach K.W. (2003). The V-type H^+^-ATPase in malpighian tubules of *Aedes aegypti*: Localization and activity. J. Exp. Biol..

[38] Dow J.A. (1999). The multifunctional *Drosophila melanogaster* V-ATPase is encoded by a multigene family. J. Bioenerg. Biomembr..

[39] Davies S.A., Goodwin S.F., Kelly D.C., Wang Z., Sözen M.A., Kaiser K., Dow J.A. (1996). Analysis and inactivation of vha55, the gene encoding the vacuolar ATPase B-subunit in *Drosophila melanogaster* reveals a larval lethal phenotype. J. Biol Chem..

[40] Lee S.-K., Li W., Ryu S.-E., Rhim T., Ahnn J. (2010). Vacuolar (H^+^)-ATPases in *Caenorhabditis elegans*: What can we learn about giant H^+^ pumps from tiny worms?. Biochim. Biophys. Acta Bioenerg..

[41] Shanbhag S.R., Müller B., Steinbrecht R.A. (1999). Atlas of olfactory organs of *Drosophila melanogaster*: 1. Types, external organization, innervation and distribution of olfactory sensilla. Int. J. Insect Morphol. Embryol..

[42] Vosshall L.B., Stocker R.F. (2007). Molecular architecture of smell and taste in Drosophila. Annu. Rev. Neurosci..

[43] Shanbhag S.R., Müller B., Steinbrecht R.A. (2000). Atlas of olfactory organs of *Drosophila melanogaster*: 2. Internal organization and cellular architecture of olfactory sensilla. Arthropod Struct. Dev..

[44] Nava Gonzales C., McKaughan Q., Bushong E.A., Cauwenberghs K., Ng R., Madany M., Ellisman M.H., Su C.-Y. (2021). Systematic morphological and morphometric analysis of identified olfactory receptor neurons in *Drosophila melanogaster*. eLife.

[45] Prelic S., Pal Mahadevan V., Venkateswaran V., Lavista-Llanos S., Hansson B.S., Wicher D. (2022). Functional interaction between *Drosophila* olfactory sensory neurons and their support cells. Front. Cell. Neurosci..

[46] Clyne P.J., Warr C.G., Freeman M.R., Lessing D., Kim J., Carlson J.R. (1999). A novel family of divergent seven-transmembrane proteins: Candidate odorant receptors in Drosophila. Neuron.

[47] Gao Q., Chess A. (1999). Identification of candidate *Drosophila* olfactory receptors from Genomic DNA sequence. Genomics.

[48] Vosshall L.B., Amrein H., Morozov P.S., Rzhetsky A., Axel R. (1999). A spatial map of olfactory receptor expression in the *Drosophila* antenna. Cell.

[49] Benton R., Vannice K.S., Gomez-Diaz C., Vosshall L.B. (2009). Variant ionotropic glutamate receptors as chemosensory receptors in Drosophila. Cell.

[50] Neuhaus E.M., Gisselmann G., Zhang W., Dooley R., Störtkuhl K., Hatt H. (2005). Odorant receptor heterodimerization in the olfactory system of *Drosophila melanogaster*. Nat. Neurosci..

[51] Vosshall L.B., Hansson B.S. (2011). A Unified Nomenclature System for the Insect Olfactory Coreceptor. Chem. Senses.

[52] Wicher D., Miazzi F. (2021). Functional properties of insect olfactory receptors: Ionotropic receptors and odorant receptors. Cell Tissue Res..

[53] Ni L. (2021). The structure and function of ionotropic receptors in *Drosophila*. Front. Mol. Neurosci..

[54] Abuin L., Bargeton B., Ulbrich M.H., Isacoff E.Y., Kellenberger S., Benton R. (2011). Functional architecture of olfactory ionotropic glutamate receptors. Neuron.

[55] Sato K., Pellegrino M., Nakagawa T., Nakagawa T., Vosshall L.B., Touhara K. (2008). Insect olfactory receptors are heteromeric ligand-gated ion channels. Nature.

[56] Wicher D., Schäfer R., Bauernfeind R., Stensmyr M.C., Heller R., Heinemann S.H., Hansson B.S. (2008). *Drosophila* odorant receptors are both ligand-gated and cyclic-nucleotide-activated cation channels. Nature.

[57] Dow J.A.T. (1995). V-ATPases in insects. Organellar Proton-ATPases.

[58] Shiao M.-S., Fan W.-L., Fang S., Lu M.-Y.J., Kondo R., Li W.-H. (2013). Transcriptional profiling of adult *Drosophila* antennae by high-throughput sequencing. Zool. Stud..

[59] Menuz K., Larter N.K., Park J., Carlson J.R. (2014). An RNA-Seq screen of the *Drosophila* antenna identifies a transporter necessary for ammonia detection. PLoS Genet..

[60] Li H., Janssens J., De Waegeneer M., Kolluru S.S., Davie K., Gardeux V., Saelens W., David F.P.A., Brbić M., Spanier K. (2022). Fly Cell Atlas: A single-nucleus transcriptomic atlas of the adult fruit fly. Science.

[61] Chen W., Gardeux V., Meireles-Filho A., Deplancke B. (2017). Profiling of single-cell transcriptomes. Curr. Protoc. Mouse Biol..

[62] Okada R., Awasaki T., Ito K. (2009). Gamma-aminobutyric acid (GABA)-mediated neural connections in the *Drosophila* antennal lobe. J. Comp. Neurol..

[63] Jain K., Stieber R., Kaltofen S., Hansson B.S., Wicher D. (2023). A new *Drosophila melanogaster* fly that expresses GFP-tagged Orco. Front. Ecol. Evol..

[64] Nicolaï L.J., Ramaekers A., Raemaekers T., Drozdzecki A., Mauss A.S., Yan J., Landgraf M., Annaert W., Hassan B.A. (2010). Genetically encoded dendritic marker sheds light on neuronal connectivity in *Drosophila*. Proc. Natl. Acad. Sci. USA.

[65] Halty-deLeon L., Pal Mahadevan V., Wiesel E., Hansson B.S., Wicher D. (2024). Response plasticity of *Drosophila* olfactory sensory neurons. Int. J. Mol. Sci..

[66] Chintapalli V.R., Wang J., Herzyk P., Davies S.A., Dow J.A.T. (2013). Data-mining the FlyAtlas online resource to identify core functional motifs across transporting epithelia. BMC Genom..

[67] Allan A.K., Du J., Davies S.A., Dow J.A.T. (2005). Genome-wide survey of V-ATPase genes in Drosophila reveals a conserved renal phenotype for lethal alleles. Physiol. Genom..

[68] Mohapatra P., Menuz K. (2019). Molecular profiling of the *Drosophila* antenna reveals conserved genes underlying olfaction in insects. G3 Genes Genom. Genet..

[69] Ganguly A., Chandel A., Turner H., Wang S., Liman E.R., Montell C. (2021). Requirement for an otopetrin-like protein for acid taste in *Drosophila*. Proc. Natl. Acs. Sci. USA.

[70] Guo Y., Wang Z., Carter A., Kaiser K., Dow J.A.T. (1996). Characterisation of vha26, the Drosophila gene for a 26 kDa E-subunit of the vacuolar ATPase. Biochim. Biophys. Acta Bioenerg..

[71] Chung Y.D., Zhu J., Han Y.-G., Kernan M.J. (2001). NompA encodes a PNS-specific, ZP domain protein required to connect mechanosensory dendrites to sensory structures. Neuron.

[72] Barolo S., Walker R.G., Polyanovsky A.D., Freschi G., Keil T., Posakony J.W. (2000). A notch-independent activity of suppressor of hairless is required for normal mechanoreceptor physiology. Cell.

